# Engagement With HIV and COVID-19 Prevention: Nationwide Cross-sectional Analysis of Users on a Geosocial Networking App

**DOI:** 10.2196/38244

**Published:** 2022-09-22

**Authors:** Thomas W Gaither, John T Sigalos, Raphael J Landovitz, Jesse N Mills, Mark S Litwin, Sriram V Eleswarapu

**Affiliations:** 1 Department of Urology David Geffen School of Medicine at UCLA University of California, Los Angeles Los Angeles, CA United States; 2 Division of Infectious Diseases, Department of Medicine David Geffen School of Medicine at UCLA University of California, Los Angeles Los Angeles, CA United States; 3 Center for Clinical AIDS Research & Education David Geffen School of Medicine at UCLA University of California, Los Angeles Los Angeles, CA United States; 4 Department of Health Policy & Management UCLA Fielding School of Public Health University of California, Los Angeles Los Angeles, CA United States; 5 School of Nursing University of California, Los Angeles Los Angeles, CA United States

**Keywords:** geosocial networking apps, pre-exposure prophylaxis, vaccination, rural, men who have sex with men, surveillance, digital surveillance, COVID-19, digital application, geosocial network, public health, surveillance platform, health platform, mobile health

## Abstract

**Background:**

Geosocial networking (GSN) apps play a pivotal role in catalyzing sexual partnering, especially among men who have sex with men.

**Objective:**

To quantify the prevalence and disparities in disclosure of pre-exposure prophylaxis (PrEP) use and COVID-19 vaccination among GSN app users, mostly men who have sex with men, in the United States.

**Methods:**

Web-based Grindr profiles from the top 50 metropolitan areas as well as the 50 most rural counties in the United States by population were randomly sampled. Grindr provides an option to disclose current PrEP use (HIV positive, HIV negative, or HIV negative with PrEP use). The free text in all profiles was analyzed, and any mention of COVID-19 vaccination was recorded. Multivariable logistic regression to assess independent associations with PrEP disclosure and COVID-19 vaccination was performed. Imputation analyses were used to test the robustness of the results.

**Results:**

We evaluated 1889 urban and 384 rural profiles. Mean age among urban profiles was 32.9 (SD 9.6) years; mean age among rural profiles was 33.5 (SD 12.1) years (*P*=.41). Among the urban profiles, 16% reported being vaccinated against COVID-19 and 23% reported PrEP use compared to 10% and 8% in rural profiles, respectively (*P*=.002 and *P*<.001, respectively). Reporting COVID-19 vaccination (adjusted odds ratio [aOR] 1.7, 95% CI 1.2-2.4), living in an urban center (aOR 3.2, 95% CI 1.8-5.7), and showing a face picture as part of the Grindr profile (aOR 4.0, 95% CI 2.3-7.0) were positively associated with PrEP disclosure. Self-identified Black and Latino users were less likely to report PrEP use (aOR 0.6, 95% CI 0.4-0.9 and aOR 0.5, 95% CI 0.4-0.9, respectively). Reporting PrEP use (aOR 1.7, 95% CI 1.2-2.4), living in an urban center (aOR 2.5, 95% CI 1.4-4.5), having a “discreet” status (aOR 1.6, 95% CI 1.0-2.5), and showing a face picture (aOR 2.7, 95% CI 1.5-4.8) were positively associated with reporting COVID-19 vaccination on their profile. Users in the southern United States were less likely to report COVID-19 vaccination status than those in the northeast United States (aOR 0.6, 95% CI 0.3-0.9).

**Conclusions:**

Variations in PrEP disclosure are associated with race, whereas COVID-19 vaccination disclosure is associated with geographic area. However, rural GSN users were less likely to report both PrEP use and COVID-19 vaccination. The data demonstrate a need to expand health preventative services in the rural United States for sexual minorities. GSN platforms may be ideal for deployment of preventative interventions to improve access for this difficult-to-reach population.

## Introduction

Pre-exposure prophylaxis (PrEP) is a highly effective and safe strategy to prevent the acquisition of HIV [1-3]. The efficacy of oral PrEP is dependent on adherence; a long-acting injectable PrEP agent was recently shown to be superior to daily oral PrEP and received US Food and Drug Administration approval on December 21, 2021 [4]. Despite these advances, men who have sex with men continue to represent 65% of new HIV diagnoses in 2019 in the United States [5]. Among this group, Black or African American and Hispanic or Latino men who have sex with men are disproportionately affected, especially within the southern United States [5]. These disparities in HIV transmission reflect many complex, psychosocial phenomena, including insular networks, social and HIV-related stigma, differential access to medical services, poverty, housing instability, racism, victimization, trauma, and discrepant PrEP awareness, access, and advocacy [6,7]. Although public disclosure of PrEP use within the men who have sex with men community may serve as a mechanism of educating others and serve as confirmation of an HIV-negative status, concerns about stigma or being labeled as sexually promiscuous limit widespread disclosure [8].

Lifetime usage of geosocial networking (GSN) apps among men who have sex with men is increasingly common, with Grindr being the frequently used by this population in the United States [9]. In a survey of adolescent men who have sex with men, 53% of them reported using apps specific to this population to meet partners for sexual intercourse [10]. Grindr allows users to locate other users within close proximity and is recognized as a “hookup” app, indicating that users seek casual, often anonymous sexual partners. More than twice as many Grindr-using men who have sex with men report 5 or more partners within the past 12 months than those who do not use the app (60% vs 27%, respectively) [11]. Many GSN apps, including Grindr, allow disclosure of HIV and PrEP status on user profiles, which may contribute to sero-sorting behaviors [12]. In fact, many users judge trustworthiness and perceived risk by observing others’ profile characteristics. Lack of pictures, minimal bios, grammatically incorrect bios, or lack of linked social media accounts are viewed as less trustworthy [13]. In a nationwide survey of urban Grindr profiles, 18.1% of users reported PrEP use, and 61% reported their HIV status [14]. PrEP disclosure was 40% less common among Black users than among White users and 30% less common among users in the southern United States than in the northeastern United States [14]. These disparities in PrEP disclosures on GSN apps mirror those reported by the Centers for Disease Control and Prevention (CDC) and, as such, may provide a real-world avenue for epidemiological surveillance.

Since the emergence of SARS-CoV-2 (the virus causing COVID-19) in 2019, HIV prevention and sexually transmitted infection (STI)–testing services have been disrupted [15]. Additionally, the pandemic has impacted sexual behavior by mostly reducing the number of sexual partners among men who have sex with men and has caused some to self-discontinue PrEP entirely or to selectively skip doses [16,17]. However, many men who have sex with men did not change sexual practices during the pandemic [18]. Reported rates of condomless anal sex with casual partners have declined; however, the association between drug use and risky sexual behavior increased [19]. Despite variations in overall sexual activity, the pandemic may be interrupting access to antiretroviral therapy, which has implications for HIV transmission [20,21]. Since implementation of social distancing and widespread closures of bars and clubs, usage of GSN apps specifically among men who have sex with men has increased, and 37% of men who have sex with men who participated in a web-based survey (N=2562) reported spending more time on apps searching for sexual partners than in prepandemic times [18]. Others have turned to GSN apps for social connectivity during lonely quarantine periods [22]. Overall, the COVID-19 pandemic has shifted sexual behavior, and GSN apps appear to play a pivotal role in catalyzing sexual partnering.

Any close contact, including but not limited to sexual intercourse, increases the risk of COVID-19 infection; vaccination against SARS-CoV-2 mitigates this risk [23,24]. How vaccination impacts the selection of sexual partners among the community of men who have sex with men remains understudied. In response to the intersection of the COVID-19 and HIV pandemics, we aimed to assess health promotional strategies, including PrEP use and COVID-19 vaccination, in web-based in a national sample of Grindr users. We sought to determine geographical and demographic trends in PrEP use and COVID-19 vaccination status disclosure to suggest geographic areas for targeting health promotion interventions. We hypothesized that men who have sex with men in rural areas would report less use of PrEP and lower disclosure of vaccination against COVID-19.

## Methods

### Sample Population

Grindr profiles from the 50 most populated metropolitan areas as well as the 50 least populated rural counties in the United States were randomly sampled [25,26]. Data collection occurred from September through December 2021 between 3 PM and 8 PM local time to ensure standardization of data collection and to eliminate time of day as a confounding factor. Each city or rural county was sampled in a random order determined by a random number generator. The number of profiles sampled from each city was proportional to the percentage of reported individuals identifying as lesbian, gay, bisexual, or transgender within that city [25]. Rural county users represented 17% of the sample, which reflects the US population [27]. Profiles were collected in the order in which they appeared within the city center or the geographic center of the rural county. A profile was created to collect the data. The profile was blank, did not contain any information, and no users were messaged. Based on previous research, we expect 18% of urban users to report PrEP use [14]. If we estimate a 6% difference as clinically significant between urban and rural users, we estimated a need to sample 2100 profiles with an 80% power and a 2-tailed α of .05.

### Exposures

All information provided within the profile was systematically abstracted into a REDCap database independently by 2 authors (TWG and JTS). REDCap is a browser-based electronic data capture software and workflow to organize and store clinical and translational research data. All geographic locations were categorized into US census region (Northeast, Midwest, South, and West) and as urban versus rural regions [27]. Available demographic characteristics included age, race and ethnicity, BMI (calculated from height and weight), gender, and relationship status. Variables were collected as presented on the app and are all self-reported. Race and ethnicity were categorized as Asian, Black, Latino, Middle Eastern, Mixed, Native American, White, South Asian, other; gender as man, cisgender man, transgender man, woman, transgender woman, nonbinary, nonconforming, queer, and crossdresser; and relationship status as single, dating, exclusive, committed, partnered, engaged, married, and open relationship. Owing to small numbers, gender was dichotomized as *cisgender male* or *gender diverse*, and relationship status was similarly dichotomized as *single* or *relationship*. Irrespective of whether the user profile presented a face picture, a body picture, other photos (neither face nor body), or no picture (blank) were abstracted. Picture status was considered a surrogate for *willingness to share*, as those who share a face picture have been found to be associated with a likelihood of reporting more data about themselves, including PrEP use [14,28].

### Outcomes

Disclosure of PrEP and vaccination status were the primary outcomes of interest. Grindr provides an option to disclose current PrEP use (HIV positive, HIV negative, or HIV negative and on PrEP). Profiles not reporting PrEP use were assumed not to be taking PrEP. The most common reason why users do not report PrEP status on GSN profiles is that they are not taking PrEP [12]. Grindr does not provide an option to report COVID-19 vaccination status. However, all free text of profiles were read and any mention of being vaccinated against COVID-19 was recorded. No profiles explicitly stated that they were not vaccinated. As such, both PrEP and vaccination outcomes in our study may represent *engagement* with HIV or COVID-19 prevention rather than actual PrEP use or vaccination status. Date of the last reported STI or HIV testing was recorded when available (n=934). Thus, our secondary outcome was the calculated time between data collection and the last STI or HIV test (in months).

### Other Variables

Other available self-reported variables were abstracted, including body type (toned, average, large, muscular, slim, or stocky), sexual position (bottom, versatile bottom, versatile, versatile top, or top), app use intent (chats, dates, friends, networking, relationship, or “right now”), and gay tribe, which is representation of certain subcultures (bear, clean-cut, daddy, discreet, jock, leather, otter, poz, rugged, trans, twink, and sober). .

### Statistical Analysis

Demographic characteristics between urban and rural profiles were compared to understand statistical correlates and to describe the population. Multivariable logistic regression was used to assess the independent factors associated with PrEP use and COVID-19 vaccination. Covariates were statistically selected (*P*<.01) on the basis of bivariate analysis. Age was included in all models owing to the availability of this variable. Because age, BMI, and race and ethnicity were not available in all profiles, the robustness of our base model was assessed with 2 imputed models—this was done to prevent the exclusion of observed data and estimate the range of selection bias [29]. The base model included only nonmissing data.

### Imputed Model A

Stepwise linear regression was used with all known variables within the data set to predict age and BMI. Actual age and BMI values were used preferentially over imputed values. Race and ethnicity were imputed using the previous known value. This approach assumes data were missing at random. A distribution of the imputed values in model A is provided in Multimedia Appendix 1.

### Imputed Model B

Data are unlikely to be missing at random. For example, older users, those with overweight or obesity, and racial minorities are more likely to experience discrimination in social environments on the internet and thus may omit this information intentionally [30]. To understand the uncertainty in the data, we used the same aforementioned imputation model but added 10 years of age to all imputed age values and +5 units to all imputed BMI values. Profiles with missing race and ethnicity data were imputed to the most common racial minority within the US census region of where the profile was found [27]. A distribution of the imputed values in model B is provided in Multimedia Appendix 1.

For our secondary outcome, the number of months since the users’ last STI or HIV test was compared among the demographic and sexual characteristics collected. Because the number of months since last testing was not normally distributed, Mann-Whitney *U* tests were used for bivariate comparisons and Kruskal-Wallis tests were performed for nominal variables. All statistical tests were 2-sided, and *P*<.05 was considered statistically significant in the final multivariable models. Analyses were completed in Stata (version 17; StataCorp).

### Ethics Approval

The institutional review board of the University of California, Los Angeles exempted the study from review as no participant protected health information was collected and no contact with users via their profiles was made.

## Results

We randomly sampled 1889 urban and 384 rural profiles. Table 1 shows the comparison of demographics by urban versus rural status. The mean age among urban profiles was 32.9 (SD 9.6) years and that among rural profiles was 33.5 (SD 12.1) years (*P*=.41). Consistent with census data, 84% of rural counties were concentrated in the western United States, whereas urban centers were distributed across US regions, (*P*<.001). Among the urban profiles, 296 (16%) reported being vaccinated against COVID-19 and 426 (23%) reported PrEP use compared to 37 (10%) and 30 (8%) in rural profiles, respectively (*P*=.002 and *P*<.001, respectively). Black users comprised 15% of urban users compared to 4% in rural areas, and Native American users comprised 0.5% in urban areas compared to 7% in rural areas. Urban users had lower BMIs than rural users (25.2 vs 27.2; *P*<.001). There were no statistically significant differences between urban and rural profiles regarding gender, relationship status, HIV disclosure status, or sexual position preference. On average, urban profiles reported STI or HIV testing within approximately 4 months versus 8 months in rural profiles (*P*<.001). Blank and “other” photos were more common among rural profiles than among urban profiles (45% vs 26%; *P*<.001).

**Table 1:** Demographic and sexual characteristics of urban versus rural Grindr profiles in the United States, 2021.

				Urban profiles (n=1889)		Rural profiles (n=384)		*P* value		Missing data in the data set, %		
**Demographic characteristics**												
	Age (years), mean (SD)^a^		32.9 (9.6)		33.5 (12.1)		.41		18.3			
	**Location in the United States, n (%)**							<.001				0
		Northeast	403 (21)		0 (0)							
		Midwest	355 (19)		22 (6)							
		South	645 (34)		41 (11)							
		West	486 (26)		321 (84)							
	Included vaccination status on profile, n (%)		296 (16)		37 (10)		.002		0			
	Taking pre-exposure prophylaxis, n (%)		426 (23)		30 (8)		<.001		0			
	**Ethnicity, n (%)^a^**							<.001		28.9		
		Asian	71 (5)		1 (2)							
		Black	203 (15)		11 (4)							
		Latino	231 (17)		38 (14)							
		Middle Eastern	15 (1)		0							
		Mixed	117 (9)		27 (10)							
		Native American	4 (0.5)		18 (7)							
		White	682 (51)		164 (61)							
		South Asian	6 (0.5)		1 (0.5)							
		Other	20 (1)		3 (1)							
	BMI, mean (SD)^a^		25.2 (3.9)		27.2 (4.2)		<.001		28.7			
	**Gender, n (%)^a^**							.73		39.9		
		Cisgender male	1051 (94)		234 (94)							
		Gender diverse	65 (6)		16 (6)							
	**Relationship status, n (%)^a^**							.54		41.9		
		Single	883 (81)		185 (79)							
		Relationship	205 (19)		48 (21)							
	Time until last test for sexually transmitted infections (months), median (IQR)^a^		4 (2-9)		8 (3-14)		<.001		58.9			
	Indicated HIV status on profile, n (%)		1148 (61)		213 (55)		.05		0			
	**Picture status, n (%)**							<.001		0		
		Face picture	1000 (53)		150 (39)							
		Body picture	399 (21)		60 (16)							
		Random photo	88 (5)		59 (15)							
		Blank	402 (21)		115 (30)							
**Sexual characteristics**												
	**Sexual position, n (%)^a^**							.24		34.1		
		Bottom	211 (18)		58 (20)		.24					
		Vers bottom	146 (12)		38 (13)							
		Vers	384 (32)		104 (36)							
		Vers top	178 (15)		37 (13)							
		Top	287 (24)		54 (19)							
	**Grindr tribe, n (%)**										0	
		Bear	88 (5)		29 (8)		.02					
		Clean-cut	172 (9)		42 (11)		.26					
		Daddy	143 (8)		34 (9)		.39					
		Discreet	236 (12)		71 (18)		.002					
		Geek	120 (6)		18 (5)		.21					
		Jock	177 (9)		27 (7)		.14					
		Leather	32 (2)		4 (1)		.35					
		Otter	87 (5)		16 (4)		.71					
		Poz	11 (1)		1 (0)		.43					
		Rugged	55 (3)		22 (6)		.01					
		Trans	60 (3)		14 (4)		.64					
		Twink	103 (5)		14 (4)		.14					
		Sober	15 (1)		4 (1)		.63					
	**Response to *Looking for*, n (%)**										0	
		Chats	749 (40)		167 (43)		.16					
		Dates	658 (35)		118 (31)		.12					
		Friends	875 (46)		184 (48)		.57					
		Networking	360 (19)		73 (19)		.98					
		Relationship	458 (24)		86 (22)		.44					
		Right now	952 (50)		223 (58)		.01					
	**Response to *Meet at*, n (%)**										0	
		My place (host)	451 (24)		96 (25)		.64					
		Your place (travel)	582 (31)		145 (38)		.01					
		Bar	299 (16)		60 (16)		.92					
		Coffee shop	295 (16)		65 (17)		.52					
		Restaurant	246 (13)		52 (14)		.78					

^a^Missing data excluded.

There were no significant differences in most Grindr tribes among urban versus rural users. There were more “discreet” users in rural areas than in urban areas (18% vs 12%; *P*=.002). There were more rural users who were using the app for the intent of immediate sexual partnering (ie, “right now”) compared to urban users (58% vs 50%; *P*=.01). Additionally, more rural users indicated a preference to meet at someone else’s location than did urban users (38% vs 31%; *P*=.01).

Table 2 shows the results of the multivariable model and imputation analyses. Reporting COVID-19 vaccination status (adjusted odds ratio [aOR] 1.7, 95% CI 1.2-2.4), living in an urban center (aOR 3.2, 95% CI 1.8-5.7), and showing a face picture (aOR 4.0, 95% CI 2.3-7.0) were positively associated with PrEP use. Self-identified Black and Latino users were less likely to report PrEP use (aOR 0.6, 95% CI 0.4-0.9 and aOR 0.5, 95% CI 0.4-0.9, respectively). All of these associations remained in the sensitivity analyses. Point estimates in the base model and model A of the association between BMI and PrEP use were not statistically significant; however, in model B, users with increasing BMI were negatively associated with PrEP use (aOR 0.85, 95% CI 0.74-0.97).

**Table 2:** Multivariable analysis of raw data and imputation models of engagement with the use of pre-exposure prophylaxis (PrEP) and COVID-19 vaccination indicated on Grindr profiles, 2021.

		PrEP use			COVID-19 vaccination status		
		PrEP use (n=1107), adjusted odds ratio (aOR) (95% CI)	Imputed model A^a^ (n=2273), aOR (95% CI)	Imputed model B^b^ (n=2273), aOR (95% CI)	COVID-19 vaccination (n=1084), aOR (95% CI)	Imputed model A^a^ (n=2255), aOR (95% CI)	Imputed model B^b^ (n=2258), aOR (95% CI)
Age (every 10 years)		0.9 (0.8-1.1)	1.0 (0.9-1.1)	1.0 (0.9-1.1)	1.1 (0.9-1.3)	1.2 (1.0-1.3)^c^	1.2 (1.1-1.4)^c^
PrEP use		—^d^	—	—	1.7 (1.2-2.4)^c^	1.8 (1.4-2.4)^c^	1.9 (1.4-2.5)^c^
Vaccinated		1.7 (1.2-2.4)^c^	1.9 (1.4-2.4)^c^	1.9 (1.4-2.5)^c^	—	—	—
Living in urban centers		3.2 (1.8-5.7)^c^	2.9 (1.9-4.4)^c^	2.8 (1.8-4.3)^c^	2.5 (1.4-4.5)^c^	2.2 (1.5-3.4)^c^	2.2 (1.4-3.3)^c^
**Region**							
	Northeast	1.0 (referent)	1.0 (referent)	1.0 (referent)	1.0 (referent)	1.0 (referent)	1.0 (referent)
	Midwest	0.9 (0.5-1.4)	0.8 (0.6-1.2)	0.8 (0.6-1.2)	1.1 (0.6-1.8)	1.3 (0.9-2.0)	1.2 (0.8-1.8)
	South	0.8 (0.5-1.2)	1.0 (0.7-1.3)	1.0 (0.8-1.4)	0.6 (0.3-0.9)^c^	0.7 (0.4-0.9)^c^	0.6 (0.4-0.9)^c^
	West	0.8 (0.5-1.3)	0.9 (0.7-1.2)	0.9 (0.7-1.3)	1.3 (0.8-2.1)	1.5 (1.0-2.2)^c^	1.5 (1.0-2.2)^c^
**Race and ethnicity**							
	Asian	1.0 (0.5-1.8)	1.2 (0.7-1.8)	0.9 (0.6-1.6)	0.7 (0.3-1.7)	1.1 (0.6-1.9)	1.2 (0.6-2.2)
	Black	0.6 (0.4-0.9)^c^	0.6 (0.4-0.9)^c^	0.6 (0.4-0.8)^c^	1.3 (0.8-2.1)	1.0 (0.7-1.5)	1.1 (0.8-1.6)
	Latino	0.6 (0.4-0.9)^c^	0.7 (0.5-0.9)^c^	0.7 (0.4-0.9)^c^	0.8 (0.5-1.4)	0.9 (0.6-1.3)	0.8 (0.6-1.1)
	Middle eastern	0.4 (0.1-2.0)	0.9 (0.3-2.8)	0.6 (0.2-2.3)	—^e^	—^e^	—^e^
	Mixed	0.8 (0.5-1.3)	0.9 (0.6-1.3)	0.8 (0.5-1.2)	1.2 (0.7-2.0)	1.0 (0.6-1.5)	1.1 (0.7-1.8)
	Native American	1.4 (0.4-5.9)	0.9 (0.3-2.5)	0.9 (0.2-3.3)	1.1 (0.2-5.3)	1.3 (0.5-3.4)	1.5 (0.5-4.7)
	South Asian	0.6 (0.1-6.6)	0.4 (0.1-2.0)	0.4 (0-3.1)	1.6 (0.2-16)	2.0 (0.5-7)	2.7 (0.5-15)
	Other	1.7 (0.4-6.3)	1.5 (0.7-3.5)	2.0 (0.8-5.0)	—	0.3 (0-1.4)	0.2 (0-1.4)
	White	1.0 (referent)	1.0 (referent)	1.0 (referent)	1.0 (referent)	1.0 (referent)	1.0 (referent)
BMI (every 5 points)		1.0 (0.8-1.2)	1.0 (0.8-1.1)	0.9 (0.7-0.9)^c^	1.2 (0.9-1.4)	1.3 (1.1-1.5)^c^	1.2 (1.0-1.3)^c^
Discreet		1.0 (0.7-1.6)	1.2 (0.9-1.7)	1.2 (0.8-1.6)	1.6 (1.0-2.5)^c^	1.6 (1.1-2.3)^c^	1.6 (1.1-2.3)^c^
**Picture status**							
	Face	4.0 (2.3-7.0)^c^	3.7 (2.6-5.2)^c^	3.3 (2.3-4.7)^c^	2.7 (1.5-4.8)^c^	3.2 (2.1-4.7)^c^	3.2 (2.2-4.8)^c^
	Body only	2.3 (1.2-4.4)^c^	2.3 (1.5-3.4)^c^	2.1 (1.4-3.1)^c^	1.6 (0.8-3.2)	2.2 (1.4-3.4)^c^	2.2 (1.4-3.4)^c^
	Random photo	2.0 (0.8-5.5)	0.9 (0.4-1.8)	0.8 (0.4-1.7)	1.8 (0.7-5.0)	2.0 (1.1-3.8)^c^	1.9 (1.0-3.6)^c^
	Blank	1.0 (referent)	1.0 (referent)	1.0 (referent)	1.0 (referent)	1.0 (referent)	1.0 (referent)

^a^Imputed model was generated through stepwise regression of known values (Multimedia Appendix 1); race and ethnicity were imputed on the basis of previous value, assuming data are missing at random.

^b^Imputed model was generated with nonrandom missing values; imputed age+10, imputed BMI +5, and race and ethnicity imputed to most common racial minority within the US census region.

^c^Boldfaced values indicate significant values at *P*<.05.

^d^Not determined.

^e^In the imputed model, none of the 15 Middle Eastern users were vaccinated and thus excluded from the model.

Reporting PrEP use (aOR 1.7, 95% CI 1.2-2.4), living in an urban center (aOR 2.5, 95% CI 1.4-4.5), having a “discreet” status (aOR 1.6, 95% CI 1.0-2.5), and showing a face picture (aOR 2.7, 95% CI 1.5-4.8) were positively associated with report of COVID-19 vaccination status on their profiles. Users in the southern United States were less likely to report being vaccinated (aOR 0.6, 95% CI 0.3-0.9) than those in the northeast United States. These associations remained in the imputation models. In both imputation models, users in the western United States were more likely to report being vaccinated (aOR 1.5, 95% CI 1.0-2.2) than those in the northeast United States, and users with increasing BMI were more likely to report vaccination (aOR 1.6, 95% CI 1.1-2.3). Model accuracy information is summarized in Table 3. Overall, the model is highly specific and poorly sensitive. Accuracy ranged from 73.9% to 85.3%.

**Table 3:** Accuracy of the models.

	Use of pre-exposure prophylaxis (PrEP)				COVID-19 vaccination status		
	PrEP use (n=1107)	Imputed model A (n=2273)	Imputed model B (n=2273)	COVID-19 vaccination (n=1084)		Imputed model A (n=2255)	Imputed model B (n=2258)
Sensitivity, %	0.7	0	0.4	1.1		0.9	0.6
Specificity, %	98.7	100	99.5	99.9		99.9	99.9
Correctly classified, %	73.9	79.9	79.6	82.5		85.3	85.3

Table 4 shows the time (in months) since the last STI or HIV test. The median time since the last STI or HIV test for the whole sample was 4 (IQR 2-10) months. Notable differences in the timing of the last STI or HIV test include the following: living in an urban versus rural center (4 months vs 8 months; *P*<.001); reporting versus not reporting PrEP use (3 months vs 6 months; *P*<.001), and BMI category (4 months: users with normal weight, 5 months: users with overweight, and 6 months: users with obesity; *P*=.003).

**Table 4:** Time since last test for sexually transmitted infections or HIV in our nationwide sample of Grindr users, 2021.

			Time (months), median (IQR)		*P* value^a^
**Age group (years)^b^**					.03
	≤30	4 (2-9)			
	>30	4 (2-11)			
**Urbanicity**					<.001
	Urban	4 (2-9)			
	Rural	8 (3-14)			
**Location in the United States**					.53
	Northeast	4 (2-8)			
	Midwest	4 (2-10)			
	South	4 (2-9)			
	West	5 (2-11)			
**COVID-19 vaccination status**					.02
	Yes	3 (2-7)			
	No	4 (2-11)			
**Status of use of pre-exposure prophylaxis**					<.001
	Yes	3 (1-5)			
	No	6 (3-13)			
**Ethnicity**					.35
	Asian	5 (2-12)			
	Black	3 (2-7)			
	Latino	6 (2-12)			
	Middle Eastern	6 (4-10)			
	Mixed	4 (2-10)			
	Native American	4 (2-10)			
	White	4 (2-10)			
	South Asian	5 (5-9)			
	Other	13 (4-15)			
**BMI**					.003
	Normal weight	4 (2-9)			
	Overweight	5 (2-10)			
	Obese	6 (3-16)			
**Gender**					.01
	Cisgender male	4 (2-10)			
	Gender diverse	2 (1-7)			
**Relationship status**					.35
	Single	4 (2-10)			
	In a relationship	4 (2-11)			
**HIV status on profile**					.18
	Yes	4 (2-10)			
	No	6 (2-12)			
**Picture status**					.10
	Face picture	4 (2-10)			
	Body picture	4 (2-9)			
	Random photo	7 (3-14)			
	Blank	4 (2-9)			
**Sexual position**					.10
	Bottom	4 (2-9)			
	Vers bottom	3 (2-8)			
	Vers	4 (2-10)			
	Vers top	4 (2-9)			
	Top	5 (2-12)			

^a^All *P* values are nonparametric tests owing to the nonnormal distribution of time since the last test for sexually transmitted infection.

^b^31 years is the median age of the sample.

## Discussion

### Principal Findings

This study provides comparative estimates of engagement in preventative health within a large nationwide sample of individuals in the United States, mostly men who have sex with men, on one GSN app during the COVID-19 pandemic. The study highlights several important differences in infection prevention behaviors between urban and rural app users. After adjustment, urban users are approximately 3 times as likely to disclose PrEP usage (aOR 3.2) and approximately 2.5 times as likely to report being vaccinated against COVID-19 (aOR 2.5). PrEP use and vaccination were often co-reported. Additionally, rural users’ last self-reported STI or HIV test was approximately 4 months delayed compared to that among urban users. Race and ethnicity appear to be highly associated with PrEP disclosure, whereas geography but not race or ethnicity appear to be associated with reporting of COVID-19 vaccination status.

Rural men who have sex with men experience substantial stigma and lack robust access to HIV preventative care [31-34]. Our results corroborate this experience in a real-world setting of users on GSN app specific to men who have sex with men, and demonstrate the need to expand preventive health services to these populations—in fact, the digital fluency of these users in leveraging a GSN-based app for sexual partnership suggests that such platforms may be ideal for deployment of interventions to support preventive care among rural populations of men who have sex with men. Internet-based HIV preventative messaging to rural men who have sex with men has been shown to be acceptable and efficacious [35]. Large-scale implementation of such programs is lacking. GSN apps may provide not only avenues for understanding spatial distribution of rural men who have sex with men but also provide a private arena to deliver care, which has been reported as an acceptable platform to deliver health information and services [36-38].

Rural users were more likely to not show their faces on their profiles and to report being “discreet.” Men who identify as discreet primarily seek sexual partners on the internet as it is seen as socially safer and can provide anonymity [39]. Potential reasons for discretion include stigma, internalized homophobia, or being in a relationship [39]. HIV-related stigma may increase the likelihood of risky sexual behavior and mental health or substance use disorders [40]. Only 8% of rural users in our study reported PrEP use, and the median time since the last STI or HIV test among rural users was 8 months. This may reflect barriers to STI HIV testing during the pandemic and more durable limitations to HIV preventative services in these areas. The rise of telemedicine access during the pandemic may serve to enfranchise the historically underserved rural population of men who have sex with men, who may benefit from improved web-based STI and HIV prevention services [33]. In response to the pandemic, many clinics for sexual minorities have developed telemedicine infrastructure for continued STI or HIV prevention services [41,42]. Removing barriers such as access and privacy concerns may improve screening and increase PrEP uptake among rural men who have sex with men [43].

Despite controlling for age, region, living in urban versus rural centers, BMI, picture status, and discreetness, Black and Latino users were less likely to report PrEP use. In the National HIV Behavioral Surveillance data analyzed by the CDC, only 30% of Hispanic and 26% of Black men who have sex with men reported taking PrEP within the past year compared to 42% of White men who have sex with men [44]. Similar disparities were observed in the southern United States compared to other regions independent of race and ethnicity [44]. PrEP is exclusively available via prescription except within states that allow pharmacists to provide this service [45]. Barriers to PrEP for Black or Latino men who have sex with men include racism, provider bias, insurance coverage, and marginalization [46,47]. The southern United States, as a region, has unique challenges to PrEP uptake, including a geographically expansive rural population, low rates of commercial insurance coverage, lack of Medicaid expansion, social stigma, low health literacy, and low HIV risk perception [48].

Despite the COVID-19 pandemic, the overall prevalence of PrEP use among urban users increased since 2018 (18% to 23%) [14]. Worldwide survey evidence suggests that many men who have sex with men have decreased or completely stopped taking PrEP during the pandemic [16,49-51]. Discontinuing PrEP during sexually inactive periods and resuming PrEP during activities that increase the risk of HIV exposure has been termed “preventive-effective” adherence [52]. Thus, some men who have sex with men who use this strategy may still report PrEP use on their GSN profile. It is plausible that the pandemic brought more PrEP-using men who have sex with men to GSN apps to meet partners after the closure of social gathering spaces such as bars and clubs. An alternative explanation is that app users who discontinued PrEP during the pandemic did not change their PrEP status on their profile. Growing evidence suggests that PrEP status can be viewed as a surrogate for desiring condomless anal sex, and some users do not present accurate information on the internet [53,54].

Whether the veracity of health-related information on GSN apps should be verified merits further inquiry. In 2011, the adult film industry created the Performer Availability Screening Service (PASS) program, which is a database to show “work clearance” by documenting STI and HIV status. The PASS program now includes verification of COVID-19 vaccination status. Widespread implementation of a system such as the PASS program would require public health and industry collaboration as well as user buy-in.

The prevalence of COVID-19 vaccination reporting within the whole sample was low (333/2273, 14.65%). The likely main driver of the overall low prevalence is that vaccination status was abstracted from free text and therefore passively reported, in contrast to HIV or PrEP status. Actual vaccination rates have been shown to be higher among sexual minorities than among heterosexual adults (85.4% vaccinated among gay and lesbian adults vs 76.3% of heterosexual adults) [55]. Interestingly, our results show that vaccination reporting is more closely associated with geographic status rather than with race or ethnicity. Lower uptake of the vaccine in rural areas is consistent with CDC data [56]. Vaccine willingness has also been associated with political leaning, which is similarly geographically associated [57]. Although Black race has been associated with lower vaccine acceptance, this was not supported by our data [58]. However, our study may be underpowered to detect these differences. If high rates of vaccine acceptability or uptake can indeed be confirmed in this population, it may provide supportive data to advocate for vaccine-based STI prevention strategies, such as the ongoing MAGI trial leveraging the serogroup B meningococcal vaccine as a potential *Neisseria gonorrhoeae* vaccine (ClinicalTrials.gov NCT04350138) [59,60]. Our data provide further evidence that once structural barriers are decreased, public health interventions may be well received among users of GSN apps.

### Limitations

Our study has certain limitations. Users of GSN apps, especially during the pandemic, may not be generalizable to all men who have sex with men. We intentionally did not sample users in suburban environments to contrast the stark geographical effects between urban and rural users. As the pandemic has shifted sexual behavior, not all users are sexually active. Important confounding variables such as education and income, which strongly impact health care access and uptake, were not available in the data set, and indeed these may be central drivers of the observed findings. All variables are self-reported and may be prone to social desirability bias. Our main outcome of interest likely represents engagement with health prevention rather than actual PrEP use or COVID-19 vaccination. We compared users who disclosed to those who did not disclose PrEP use or COVID-19 vaccination status. Young Black men who have sex with men in the southern United States report high usage of GSN apps to meet sexual partners, but Grindr was the least commonly used app [61]. The rates of PrEP disclosure among Black or Latino men who have sex with men who use other apps remain to be studied. Imputation analyses are dependent on the reasons for missing data and can significantly impact measurement error, limiting power. Overall, the modeling accuracy was adequate, but the sensitivity was extremely poor. The statistical aim of the modeling was to assess associations with our outcomes of interest and not to generate a prediction model.

### Conclusions

We demonstrate a need to expand health preventative services for sexual minorities in the rural United States. Disclosure of PrEP use and COVID-19 vaccination status on GSN app profiles mirror trends assessed by CDC survey data. Variations in PrEP disclosure are largely impacted by race, whereas COVID-19 vaccination disclosure is influenced by geographic area; however, rural GSN users were less likely to report both PrEP use and COVID-19 vaccination status. GSN platforms may be ideal for deployment of preventative interventions to improve access for this difficult-to-reach population.

## Appendix

Multimedia Appendix 1 Supplemental table.

## Abbreviations

aOR: adjusted odds ratio
CDC: Centers for Disease Control and Prevention
GSN: geosocial networking
PASS: Performer Availability Screening Service
PrEP: pre-exposure prophylaxis
STI: sexually transmitted infection
