# Supplementary material for: Engagement With HIV and COVID-19 Prevention: Nationwide Cross-sectional Analysis of Users on a Geosocial Networking App
Source: J Med Internet Res. 2022 Sep 22;24(9):e38244. doi: 10.2196/38244 (PMC9512083; doi:10.2196/38244)
Supplement: Multimedia Appendix 1 [file jmir_v24i9e38244_app1.docx]

**Supplemental Table. Age, Race/Ethnicity, and BMI in non-missing and imputed models**

|  | Non-missing population | Model A^a^ | Model B^b^ |  |
| --- | --- | --- | --- | --- |
|  |  |  |  |  |
| Age, mean (SD) | 33.0 (10.3) | 33.2 (9.5) | 35.0 (10.3) |  |
|  |  |  |  |  |
| Ethnicity, n(%) |  |  |  |  |
| Asian | 76 (4.7) | 108 (5) | 76 (3) |  |
| Black | 214 (13) | 291 (13) | 511 (22) |  |
| Latino | 269 (17) | 363 (16) | 629 (28) |  |
| Middle Eastern | 15 (1) | 18 (1) | 15 (1) |  |
| Mixed | 144 (9) | 199 (9) | 144 (6) |  |
| Native American | 22 (1) | 35 (2) | 22 (1) |  |
| White | 846 (52) | 1215 (53) | 846 (37) |  |
| South Asian | 7 (0.5) | 13 (1) | 7 (0.5) |  |
| Other | 23 (1) | 31 (1) | 23 (1) |  |
|  |  |  |  |  |
| BMI, mean (SD) | 25.5 (4.0) | 25.6 (3.7) | 27.0 (4.3) |  |
|  |  |  |  |  |

^a^ Imputed model built off stepwise regression of known values; race/ethnicity imputed based on previous value; assuming data missing at random

^b^ Imputed model built of non-random missing values; imputed age+10, imputed BMI +5, race/ethnicity imputed to most common racial minority within US census region
